# Synthesis and Properties of Thiophene and Aniline Copolymer Using Atmospheric Pressure Plasma Jets Copolymerization Technique

**DOI:** 10.3390/polym12102225

**Published:** 2020-09-28

**Authors:** Hyo Jun Jang, Choon-Sang Park, Eun Young Jung, Gyu Tae Bae, Bhum Jae Shin, Heung-Sik Tae

**Affiliations:** 1 School of Electronic and Electrical Engineering, College of IT Engineering, Kyungpook National University, Daegu 41566, Korea; bs00201@knu.ac.kr (H.J.J.); eyjung@knu.ac.kr (E.Y.J.); doctor047@knu.ac.kr (G.T.B.); 2 Department of Electrical and Computer Engineering, College of Engineering, Kansas State University, Manhattan, KS 66506, USA; purplepcs@ksu.edu; 3 Department of Electronics Engineering, Sejong University, Seoul 05006, Korea; hahusbi@sejong.ac.kr; 4 School of Electronics Engineering, College of IT Engineering, Kyungpook National University, Daegu 41566, Korea

**Keywords:** thiophene and aniline copolymer (TAC), donor–acceptor (D–A) copolymer, conjugated copolymers (CCPs), aniline, thiophene, atmospheric pressure plasma (APP), atmospheric pressure plasma jet (APPJ)

## Abstract

This paper investigates the properties of thiophene and aniline copolymer (TAC) films deposited by using atmospheric pressure plasma jets copolymerization technique relative to various blending ratios of aniline and thiophene monomer for synthesizing the donor–acceptor conjugated copolymers. Field emission scanning electron microscopy (FE-SEM) and atomic force microscopy are utilized to measure the surface morphology, roughness and film thickness of TAC films. Structural and chemical properties of TAC films are investigated by Fourier transforms-infrared spectroscopy (FT-IR), time of flight secondary ion mass spectrometry, and X-ray photoelectron spectroscopy. FE-SEM images show that the film thickness and nanoparticles size of the TAC films increase with an addition thiophene monomer in the aniline monomer. FE-SEM, FT-IR results show that TAC films are successfully synthesized on glass substrates in all cases. The iodine doped TAC film on the Si substrate with interdigitated electrodes shows the lowest electrical resistance at blending condition of thiophene of 25%.

## 1. Introduction

In recent, donor–acceptor (D–A) conjugated copolymers (CCPs) have been widely investigated on the band gap engineering in the industrial and academic areas for flexible organic field-effect transistors, photocatalysts, polymer solar cells, and electrochromic applications [1,2,3,4]. Among of D–A CCPs, the copolymer of aniline with thiophene is known to increase the conductivity of polyaniline (PANI) and thiophene-based polymeric materials have great electrochromic properties suitable for displays such as high contrast, fast switching times, and stabilities [5,6,7]. Also, aniline acts as an electron donor, while thiophene is known as an electron acceptor in thiophene and aniline copolymer (TAC) [8]. D–A CCPs synthesis processes in many recent studies are mainly chemical or electrochemical method having several inefficiencies such as requiring a catalyst, long time process, solubility problems, and different synthesis methods depending on the substance [9,10,11,12]. In particular, for avoiding the soluble problem, the exploitation of the monomer having various substituent groups for increasing the solubility causes a loss of conductivity of the polymer [13]. In spite of these limitations, these conventional methods are occasionally unsuitable when the oxidation potential difference between substances is large such as the synthesis of aniline and thiophene [5,14]. In order to solve these inefficiencies, the synthesis using abundant radicals and ions generated by plasma is reasonable alternative instead of conventional method for synthesizing a CCPs [15,16,17,18]. In recent, C-S Park et.al. has already reported on the high-quality polymer film synthesis using by the atmospheric pressure plasma jets (APPJs) with the simple guiding tube and bluff body (GB) system [19,20]. This high quality polymer thin film synthesis is inherently due to intensified glow-like plasma produced by the APPJs using the GB system. Thanks to the GB system, rich radicals and ions which increase reactivity are maintained without quenching of ambient air. This intense glow-like plasma generated by APPJs can provide a sufficient fragmentation of monomers and generate efficiently new copolymeric products in which fragments of various monomers are covalently interlinked [19,20,21,22,23,24]. Unlike the restriction to the plasma plume of the process area in case of other plasma jet techniques, the APPJs with GB system can produce the intensified uniform plasma in the wide process space during copolymerization process [19,22]. Furthermore, the APPJs technique has some unique properties such as one-step process, cost-effective, dry process, low-temperature process, and green synthesis [17,23,24,25]. Owing to these reasons, the APPJs polymerization technique is suitable for synthesizing D–A CCPs based on the blending process of various monomers. Nonetheless, there have been no reports on the synthesis of copolymer films prepared by the APPJs polymerization technique.

Accordingly, this study carried out the synthesis of copolymerization using by the APPJs polymerization technique with blending solution of aniline and thiophene monomers. In this work, the changes in the structural and electrical properties the TAC films are examined as a function of the blending ratio between the aniline and thiophene monomers. The properties of the TAC films grown by the APPJs polymerization technique are investigated in detail by field emission scanning electron microscopy (FE-SEM), Fourier transforms infrared spectroscopy (FT-IR), atomic force microscopy (AFM), X-ray photoelectron spectroscopy (XPS), and time of flight-secondary ion mass spectrometry (ToF-SIMS). In particular, in order to identify the suitability of conductive layer with high electrical conductivity, the electrical resistances of the TAC films are measured at a room temperature (R.T.) condition by using two probe methods after ex-situ doping with iodine (I_2_).

## 2. Materials and Methods 

### 2.1. Experimental Setup

**Figure 1:**
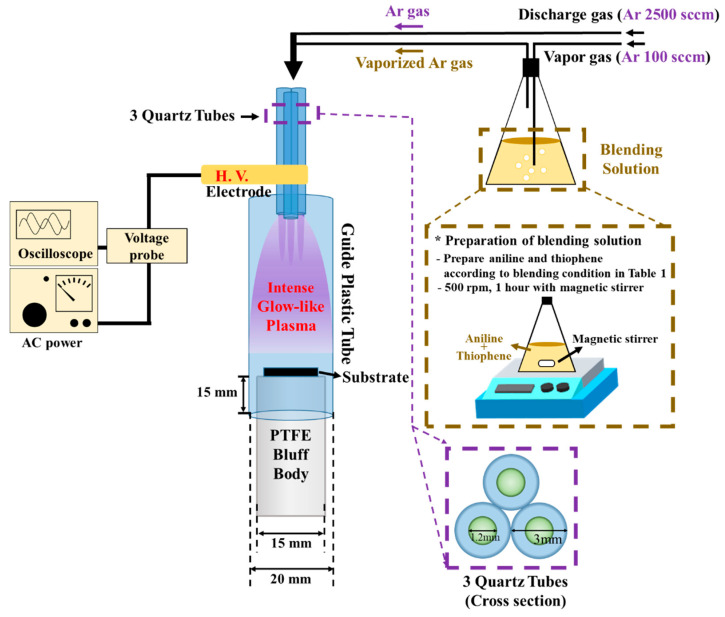
Schematic diagram of APPJs polymerization setup and preparation of blending solution employed in this study.

The APPJs device consisted of 3 quartz tube jets, a polyethylene terephthalate (PET) guide tube and, a polytetrafluoroethylene (PTFE) bluff body. The size of each quartz tube jet was 1.2 mm of an inner diameter (I.D.) and 3 mm of an outer diameter with 130 mm in length. The quartz jets were arranged in a triangle shape and wrapped with a copper tape acting as a powered electrode, 10 mm away from the tip of jets. The PET guide tube was cylindrical tube with an I.D. of 20 mm and length of 60 mm. Figure 1 describes a schematic diagram of the experimental setup. The experimental equipment of a novel APPJs polymerization system has been previously explained in detail by C-S Park et.al. [19,24]. Argon gas (99.999%, Linde korea, Pohang, Korea) flow rates of 2500 standard cubic centimeters per minute (sccm) and 100 sccm were used to generate a plasma and vaporize blended solutions, respectively. An applied power was sinusoidal voltage wave with a peak value of 11.5 kV at frequency of 26 kHz. Then, the glass and Si wafer were used as the substrates in the APPJs polymerization technique. The process time was 30 min for case I and 8 min for other cases.

### 2.2. Preparation of Blended Solution and Deposition of Copolymer Thin Film

To synthesize the TAC films using the APPJs polymerization, 40 mL of blended solutions were prepared together in a flask with respect to various ratios of aniline (Sigma-Aldrich Co., St. Louis, MO, USA) and thiophene (Sigma-Aldrich Co., St. Louis, MO, USA) monomers. To efficiently blend aniline and thiophene monomers, blended solutions were stirred at angular velocity of 500 rpm for 1 h without agglomeration. The blended solutions were used to deposit TAC films by using the APPJs polymerization method as a function of various concentrations of aniline and thiophene monomers. In addition, before depositing TAC films, a glass substrate and a Si substrate with interdigitated electrodes (1 cm × 1 cm) were prepared by an ultrasonic cleaning with an acetone, ethanol, and de-ionized water in sequence for 20 min. Then, the blended solutions of aniline and thiophene were deposited on glass substrates by APPJs polymerization method. Deposition conditions of TAC films are given in detail in Table 1.

### 2.3. Field Emission-Scanning Electron Microscopy

The surface morphology and cross-sectional images of TAC films were investigated by using Field emission-scanning electron microscopy (FE-SEM: Hitachi SU8220, Hitachi, Tokyo, Japan) with accelerated electrons at voltage and current of 3 kV and 10 mA, respectively. Prior to measurement, the TAC film on the glass was coated with conductive platinum in the vacuum chamber. In addition, in order to check the mechanical property for flexible application, the bending test was performed on TAC films deposited on polyethylene terephthalate (PET). Then, after bending test with a few times, the plane-view images of the TAC films were obtained by FE-SEM (Gemini-500, ZEISS, Oberkochen, Germany) at the Research Institute of Industrial Science and Technology (RIST; Pohang, Korea) with accelerating voltage and current of 2 kV and 10 mA, respectively.

### 2.4. Atomic Force Microscopy

The surface roughness of TAC films was performed on a non-contact mode by Atomic Force Microscopy (AFM: NanoWizard II, Brucker, Berlin, Germany) at the Korea Basic Science Institute (KBSI; Busan, Korea). All measurements were obtained under controlled room temperature. Moreover, the scanning area was 20 μm × 20 μm and scan rate was set at 1 Hz. The Bruker NanoWizard software was used for image processing and interpretation.

### 2.5. Fourier Transformation Infrared Spectroscopy

The molecular structure of the TAC film on the glass was detected by a Fourier transformation infrared spectroscopy (FT-IR: Vertex 70, Bruker, Berlin, Germany) at the Korea Basic Science Institute (KBSI; Daegu, Korea). FT-IR spectra were measured by averaging 128 scans at a wavenumber resolution of 0.6 cm^−1^ in the range from 650 to 4000 cm^−1^ using attenuated total reflection (ATR) mode.

### 2.6. X-ray Photoelectron Spectroscopy

An X-ray photoelectron spectroscopy (XPS: ESCALAB 250XI, Thermo Fisher Scientific, Waltham, MA, USA) was used to investigate surface chemical compositions and atomic ratios of TAC films on the glass. In the XPS measurement, the voltage and current of the monochromatic Al Kα X-ray source (hν = 1486.7 eV) were 15 kV and 20 mA, respectively. The area of 500 μm × 500 μm was measured under the vacuum condition of about 10^−8^ Pa. The energy scale was calibrated using the C 1s spectrum (285.0 eV). Elements present on the TAC surface were classified from XPS survey scans and quantified with Thermo Avantage software (v.5.977, Waltham) using a Shirley background. For high-resolution spectra, the constant analyzer energy modes were used at 200 eV and 50 eV to conduct the survey scan and the element scan, respectively. During the measurement of the TAC films on the glass, an additional electron gun was used to adjust the charge compensation for maintaining surface neutralization. To fit the curves for the high-resolution C 1s, N 1s, S 2p, and O 1s peaks, peak deconvolutions were analyzed by the Thermo Avantage software. Peaks were deconvoluted using Gaussian-Lorentzian peak shapes (constrained between 80 and 100% Gaussians) and the full-width at half maximum (FWHM) of each line shape was constrained between 2.0 and 3.0 eV.

### 2.7. Time of Flight-Secondary Ion Mass Spectrometry

The surface structure and composition of TAC films on the glass were examined by the time of flight-secondary ion mass spectrometry (ToF-SIMS: TOF-SIMS5, ION-TOF GmbH, Münster, Germany) with a bismuth primary-ion (Bi_3_^+^) gun source. The pressure in the ToF-SIMS chamber was maintained below 1 × 10^−9^ Torr. Bi_3_^+^ (0.5 pA) accelerated at 30 keV was used as the analysis (primary) gun. The negative-ion and positive ion mass spectra of a 500 μm × 500 μm area were acquired at a Bi_3_^+^ primary-ion beam of 30 keV.

### 2.8. Two-Probe Method

The electrical resistances of TAC films on the Si substrate with interdigitated electrodes were measured at a R.T. by a two-probe method using electrometer (FLUKE 179, FLUKE, Everett, WA, USA) after ex-situ doping with iodine (I_2_). For ex-situ I_2_ doping, samples of deposited TAC films were placed in a sealed glass container with solid I_2_ crystals of 2 g (99.99%, Sigma-Aldrich Co., St. Louis, MO, USA) for 30 min.

## 3. Results and Discussion

**Figure 2:**
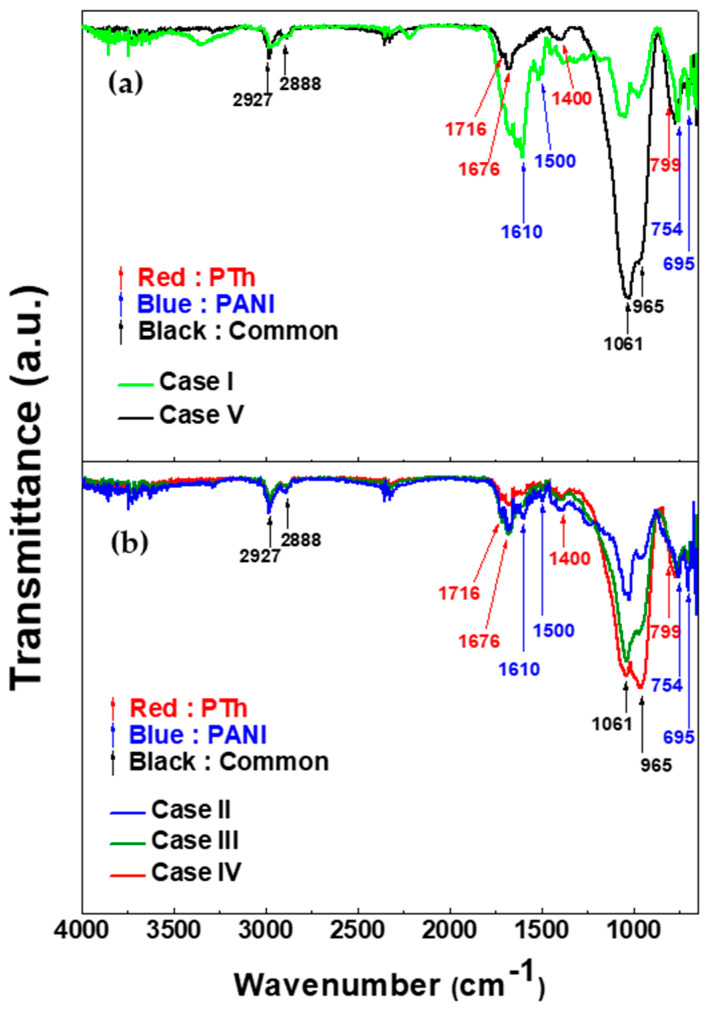
FT-IR spectra of (**a**) homopolymer films and (**b**) thiophene and aniline copolymer (TAC) films deposited on glass substrate by using atmospheric pressure plasma jets (APPJs) polymerization relative to various ratios of blended solutions of aniline and thiophene liquid monomer.

Figure 2 shows the FT-IR spectra of (a) homopolymer films and (b) TAC films deposited on glass substrate by using APPJs polymerization relative to various ratios of blended solutions of aniline and thiophene liquid monomer. In homopolymer cases (cases I and V) of Figure 2a, both cases have the common peaks at 965, 1061, 2888, 2927 cm^−1^. The peaks at 965 cm^−1^ and 1061 cm^−1^ are due to the C–H out-plane and in-plane bending vibration from aromatic ring such as benzenoid, quinoid, and thiophene ring, respectively [26,27,28,29,30]. The assigned peaks at 2888 cm^−1^ and 2927 cm^−1^ are attributed to the aliphatic of C–H stretching within polymer chain [28]. As shown in Figure 2a, polythiophene deposited by APPJs has the characteristic peaks at 799, 1400, 1676, and 1716 cm^−1^. The peak at 799 cm^−1^ is assigned the band of C−S stretching vibration in thiophene ring [28,29]. C=C stretching vibration in thiophene ring is measured at around 1400 cm^−1^. Peaks at 1676 cm^−1^ and 1716 cm^−1^ are ascribed to the C=O stretching. These carbonyl groups are formed due to the reaction of thiophene and oxygen in ambient air [8,27,30]. The characteristic peaks of polyaniline deposited by APPJs appear at 695, 754, 1500, 1610, and 1676 cm^−1^. The peaks located at 695 cm^−1^ and 754 cm^−1^ are assigned to meta- and ortho- substitutions in benzene ring, respectively [26,27]. It is expected that the TAC films prepared by APPJs polymerization are not straight structure due to the presence of these peaks. Peaks at 1500 cm^−1^ and 1610 cm^−1^ are attributed to benzenoid and quinoid rings stretching vibrations, respectively [14,26,27]. The peak at 1676 cm^−1^ can be defined to be C=N stretching vibration of quinoid ring in aniline [14,27,31,32]. In all copolymer cases (cases II, III, and IV) of Figure 2b, the absorption peaks of the TAC films clearly illustrate the characteristic peaks originating from both PANI and PTh homopolymer. In particular, the presence of the peaks at 1400, 1500, and 1610 cm^−1^ in TAC films confirms that the ring structure (thiophene ring, benzene ring, and quinoid ring) is successfully preserved in TAC films during plasma copolymerization. The chemical structures corresponding to the characteristics peaks in Figure 2a are summarized in Table 2.

**Figure 3:**
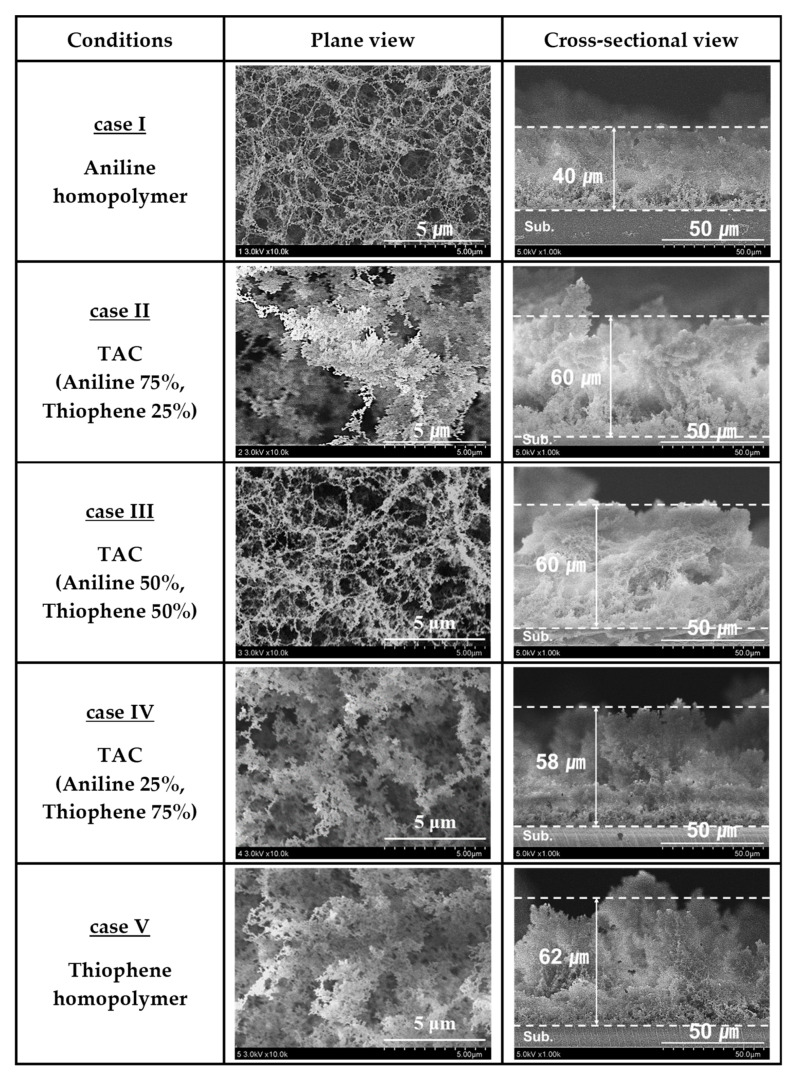
Plane and cross-sectional FE-SEM images of TAC films including PANI and PTh films deposited on glass substrates by APPJs polymerization relative to various ratios of blended solutions of aniline and thiophene monomers.

Figure 3 shows the plane and cross-sectional FE-SEM images of TAC films including PANI and PTh films deposited on glass substrates by the APPJs polymerization technique with various blending ratios of aniline and thiophene monomers. As shown in Figure 3, the aniline homopolymer film is comprised of nanoparticles and nanofibers with irregularly cross-linked networks [24,33]. When increasing the thiophene ratio, the nanoparticles grow bigger and the nanofibers are likely to be thick and dense with irregularly cross-linked networks. Moreover, the TAC films are composed of nanoparticles and nanofibers with irregularly cross-linked networks. In this case, the particle size of the TAC films is observed to be larger than that of pure PANI with increasing the thiophene ratio. Accordingly, the TAC films have a high cross-linked morphology with increasing the thiophene ratio, as shown in Figure 3.

**Figure 4:**
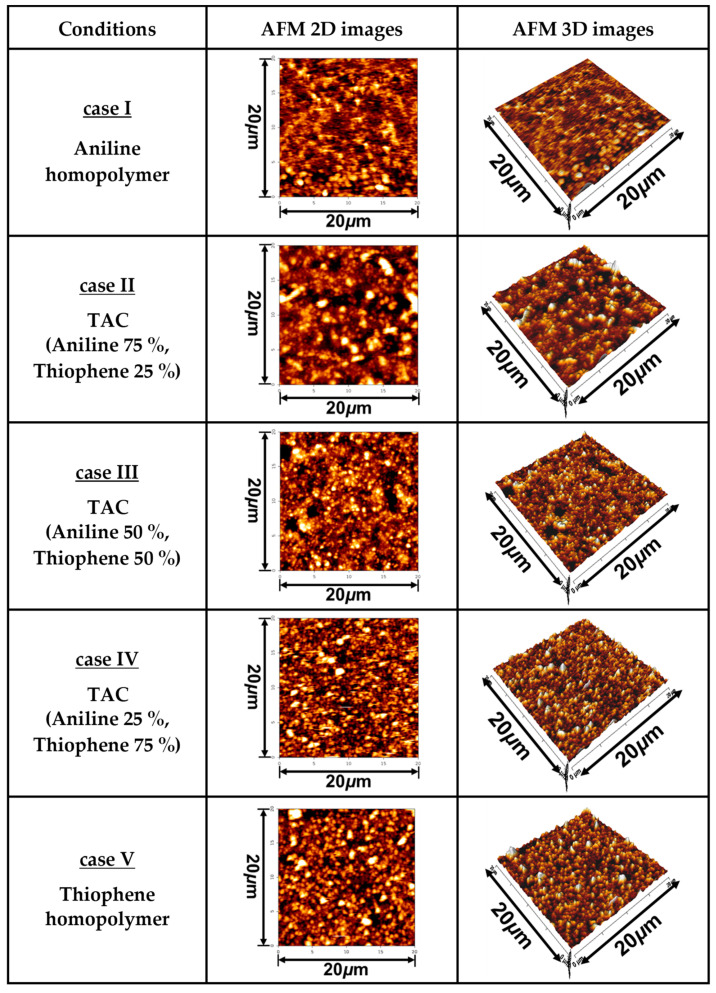
Two- (2D) and three-dimensional (3D) AFM images of TAC film surfaces grown on glass substrate by APPJs technique relative to various ratios of blended solutions of aniline and thiophene monomers.

Figure 4 show the changes in two- (2D) and three-dimensional (3D) AFM images of TAC film surfaces grown on glass substrates relative to various ratios of blended solutions of aniline and thiophene monomers. The root mean square roughness (Rq) and average roughness (Ra) obtained from the AFM images of TAC film surfaces of Figure 4 are summarized in Table 3. For homopolymer of the deposited PANI film (case I), the surface roughnesses of Rq and Ra were 8.9 nm and 5.7 nm, respectively. Plus, for homopolymer of PTh (case V), the surface roughnesses of Rq and Ra were 59.2 nm and 44.2 nm, respectively. These results illustrate that the roughness of PTh film is larger than that of PANI film. In Figure 4, the roughness of three copolymer cases (cases II, III, and IV) is shown to be ranged between the pure PANI and PTh cases.

**Figure 5:**
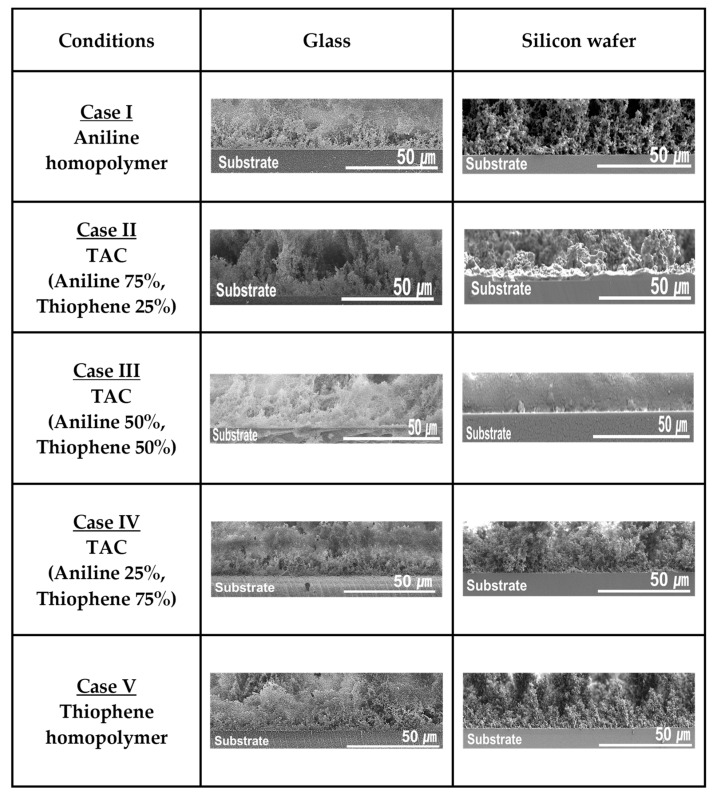
Homogenous growth property of TAC films including PANI and PTh films deposited by APPJs technique with various cases of blended solutions of aniline and thiophene liquid monomers on glass and silicon substrates.

Figure 5 shows the homogenous growth property of TAC films including PANI and PTh films grown by APPJs technique on the different substrates such as glass and silicon substrates with various cases studies of blended solutions of aniline and thiophene liquid monomers. In Figure 5, the homogenous growth property of TAC films was measured through the cross-sectional FE-SEM. For all cases, the result of Figure 5 confirms that the TAC films have a good adhesion property on both the glass and silicon substrates. Furthermore, the bending test for the TAC films grown on PET substrate is carried out to investigate the mechanical property of the TAC films. The film quality of the TAC film after bending test 10 times is observed to remain almost constant, which is monitored by the plane-view of the FE-SEM (not shown here). This good mechanical property of the TAC films is inherently due to the good adhesion property.

**Figure 6:**
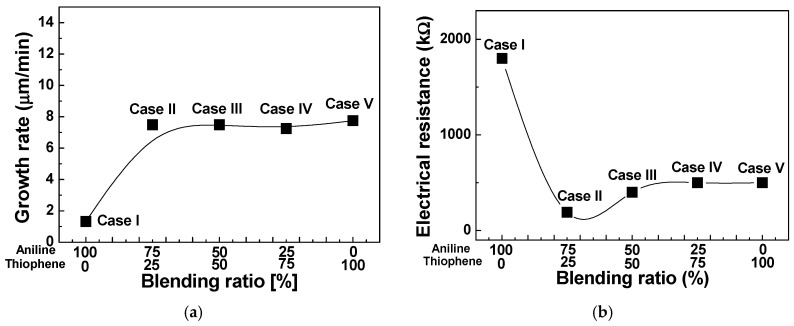
(**a**) Growth rate and (**b**) electrical resistance of TAC films deposited by APPJs polymerization relative to various ratios of blended solutions of aniline and thiophene monomers.

Figure 6a,b show the changes in the growth rate and electrical resistance for TAC films deposited by the APPJs polymerization technique relative to various blending ratios of aniline and thiophene monomers. Growth rates of pure thiophene and aniline film have 7.75 and 1.33 μm/min, respectively. As shown in Figure 6a, growth rates of the TAC film were sharply increased by adding the thiophene monomer in aniline, which was presumably because the thiophene monomer with low oxidation potential catalyzes the polymerization of aniline monomer with high oxidation potential [5,14]. Figure 6b shows the changes in the electrical resistance of I_2_ doped TAC films deposited on Si substrates of interdigitated electrodes relative to various blending ratios of aniline and thiophene monomers. For the homopolymer cases (cases I and V), the measured electrical resistance was about 500 kΩ for PTh and 1800 kΩ for PANI. The aniline homopolymer film has a high electrical resistance than the thiophene homopolymer film. For the TAC film with thiophene ratio of 25%, the electrical resistance is measured to decrease remarkably. This considerable decrease of electrical resistance (190 kΩ) is mainly based on the fact that that the TAC films would have a good connection between the donor (aniline) and acceptor (thiophene) monomers reducing a loss of charge transport pathways thanks to conjugated π-bonds and thick and dense nanofibers in the TAC film [33,34,35]. Accordingly, the change of electrical resistance depends on the morphology and a connection between the aniline and thiophene monomers of the TAC films. These experimental results confirm that there is an optimal blending condition, that is, thiophene concentration of 25% in this experiment, for improving the electrical conductivity of TAC film grown by the APPJs polymerization. As a result, we obtained that at the thiophene concentration of 25% (case II), the TAC films having a low resistance of 190 kΩ were successfully synthesized by the APPJs polymerization.

**Figure 7:**
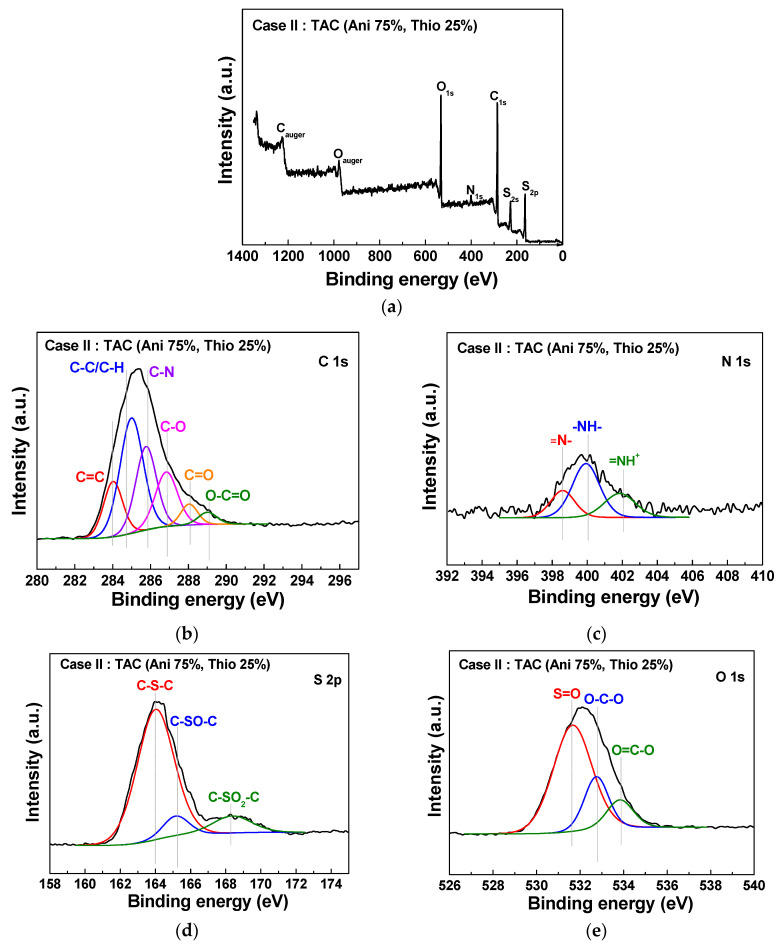
XPS survey spectra (**a**) and detailed high-resolutions with deconvolutions (**b**–**e**) of TAC films deposited on glass substrates by APPJs polymerization at blending condition of case II (aniline 75% and thiophene 25%).

**Figure 8:**
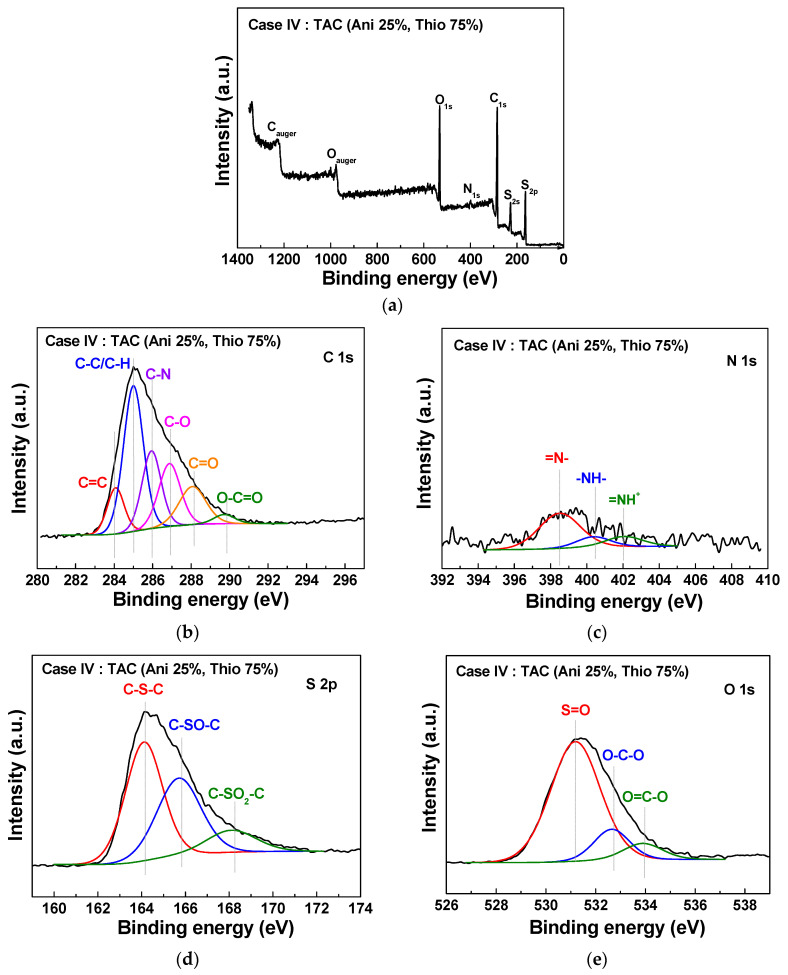
XPS survey spectra (**a**) and detailed high-resolutions with deconvolutions (**b**–**e**) of TAC films deposited on glass substrates by APPJs polymerization at blending condition of case IV (aniline 25% and thiophene 75%).

Figure 7 and Figure 8 show the XPS elemental compositions of TAC films deposited at cases II and IV, respectively. In Figure 7a and Figure 8a, the TAC films consist of a carbon, nitrogen, sulfur, and oxygen. Elemental compositions of the TAC films are summarized in Table 4. In Figure 7b and Figure 8b, component peaks of C 1s spectra around 284.1, 285.0, 285.9, 286.9, 288.1, and 289.7 eV are confirmed to be corresponding to C=C, C–C/C–H, C–N, C–O, C=O, and O–C=O, respectively [29,36,37]. In Figure 7c and Figure 8c, the N 1s spectrum is comprised of three peaks centered around 398.5, 400.4, and 402.1 eV, which correspond to the imine (=N–), amine (–NH–) structure, and protonated imine (=NH^+^), respectively [38,39]. In Figure 7d and Figure 8d, the S 2p spectrum is comprised of three peaks centered at 164.1, 165.7, and 168.1 eV, which correspond to the C–S–C, C–SO–C, and C–SO_2_–C, respectively [29,36]. In Figure 7e and Figure 8e, the O 1s spectrum is decomposed into three components at 531.2, 532.6, and 533.9 eV, which correspond to the S=O, O–C–O, and O=C–O, respectively [29,36]. The peak assignments and envelope compositions of high-resolution with deconvolutions (C 1s, N 1s, S 2p, and O 1s) of Figure 7 and Figure 8 are summarized in Table 5. The high electrical conductivity of TAC film is deeply related to the ratios of amine (–NH–) and imine (=N–) groups. The imine (=N–) functional group involves a presence of quinoid ring and quinone type thiophene ring. Also, the presence of amine (–NH–) functional group implies a linkage of benzene ring-thiophene ring or between benzene rings [8,38]. In the oxidation type doping like iodine doping, the π-conjugated system of thiophene ring and the amine (–NH–) group form the defect states of TAC films such as polaron and bipolaron. These states have an influence on the increasing of the conductivity by iodine doping [14,28,37,40,41,42]. In order to investigate the relation between the electrical conductivity and ratios of amine (–NH–) and imine (=N–), we calculated the value of the ratio of amine and imine group (–NH–/=N–), as shown in Table 5. From these results, the ratios of amine and imine group (–NH–/=N–) of cases II and IV represent 2.3 and 0.3, respectively. The higher value of 2.3 for case II implies that the TAC films grown at case II have lots of amine (–NH–) group, thereby causing the high electrical conductivity by forming the defect states such as polaron and bipolaron in TAC films through the simple iodine doping. As a result, we obtain an optimal blending condition (case II) for improving the electrical conductivity of the TAC films synthesized by the APPJs polymerization, and as such, the low resistance of TAC films with 190 kΩ is obtained at the blending ratio of 75% aniline and 25% thiophene by ex-situ iodine doping.

**Figure 9:**
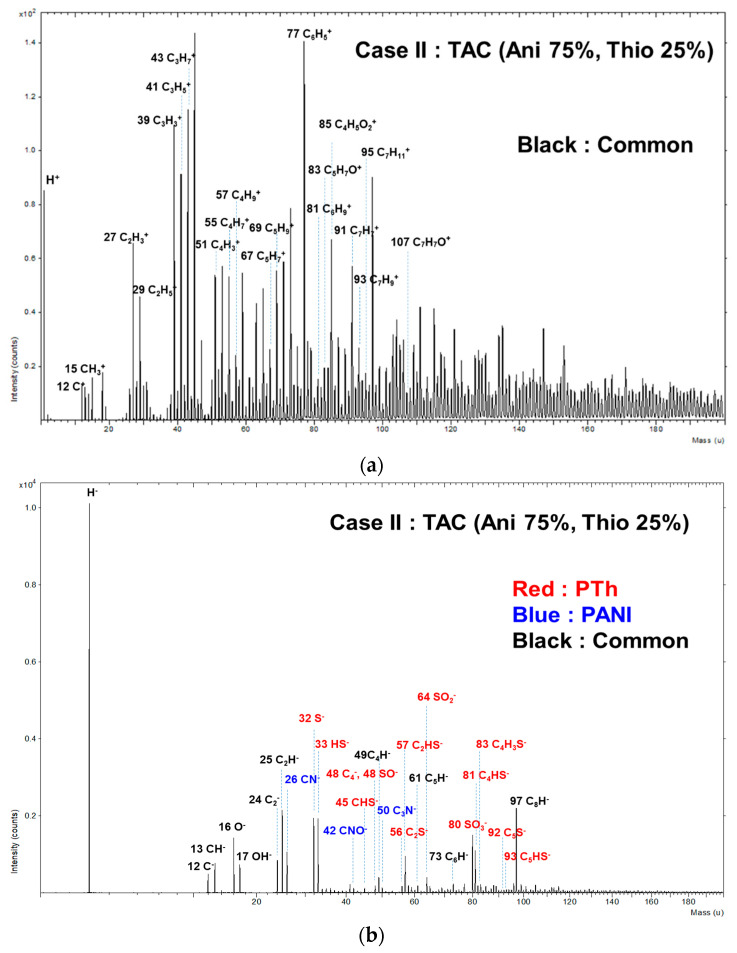
Positive-ion (**a**) and negative-ion (**b**) (0–200 amu) of ToF-SIMS on surface of TAC films deposited on glass substrates by APPJs polymerization at blending condition of case II (aniline 75% and thiophene 25%).

To support the presence of copolymer structure of TAC film grown at blending condition of case II based on the characteristics peaks of FT-IR of Figure 2b, the TAC film was measured in both the positive and negative ion modes using ToF-SIMS. Figure 9 shows the positive ion (a) and negative ion (b) with wide range from 0 to 200 amu static mass spectra when using ToF-SIMS for the TAC films deposited at case II. Several characteristic peaks detected in the positive ion mode of Figure 9a such as C_2_H_5_^+^, C_3_H_3_^+^, C_3_H_5_^+^, C_3_H_7_^+^, C_4_H_3_^+^, C_4_H_9_^+^, and C_6_H_5_^+^. C_6_H_5_^+^ would be originated from the polymer chain such as benzenoid ring of aniline. It is expected that other peaks would be typical aliphatic hydrocarbon fragments such as C_n_H_2n-3_, C_n_H_2n-1_, and C_n_H_2n+1_, arising from the PANI and PTh chain [24,37,43]. Moreover, as shown in negative ion mode of Figure 9b, the peaks corresponding to m/z = 26, 42, and 50 amu would be attributed to CN^−^, CNO^−^, and C_3_N^−^, respectively, which are likely to be peaks of PANI fragments. In Figure 9b, S^−^, HS^−^, CHS^−^, C_2_S^−^, C_2_HS^−^, SO_2_^−^, SO_3_^−^, C_4_HS^−^, and C_4_H_3_S^−^ are also found at *m*/*z* = 32, 33, 45, 56, 57, 64, 80, 81, and 83 amu, which are likely to be peaks of PTh fragments. In particular, C_4_HS^−^ and C_4_H_3_S^−^ would stem from the thiophene ring [43,44].

## 4. Conclusions

In this work, the TAC films were successfully synthesized by the APPJs polymerization technique based on various blending ratios of aniline and thiophene monomers. The properties of the TAC films including the surface morphology, roughness, and film thickness were investigated by using FE-SEM, FT-IR, XPS, ToF-SIMS, AFM, and two-probe method. It is observed that both the size and growth rates of nanoparticles of the TAC films increase with adding the thiophene monomer in aniline monomer. We find that there is an optimal blending condition (case II) for improving the electrical conductivity of the TAC films synthesized by the APPJs polymerization, and as such, the low resistance of TAC films, 190 kΩ, is obtained at the blending ratio of 75% aniline and 25% thiophene by ex-situ iodine doping in this experiment. We also check the inter-particle bonding of the TAC film grown at the blending condition of case II. This APPJs polymerization technique is expected to be a low cost, simple, and effective method for improving the electrical conductivity of copolymers with large oxidation potential difference between precursors.

## Figures and Tables

**Table 1:** Blending ratios of aniline and thiophene monomers and deposition conditions of APPJs polymerization technique.

Solution	Liquid Monomer (Aniline + Thiophene), 40 mL					
Blending conditions		case I	case II	case III	case IV	case V
	Thiophene (%)	0	25	50	75	100
	Aniline (%)	100	75	50	25	0
Ar gas for vaporization	100 sccm					
Ar gas for discharge	2500 sccm					
Voltage	23 kV					
Frequency	26 kHz					
Deposition time	8 min (30 min in case I)					
Deposition temperature	R. T.					

**Table 2:** Absorption peaks of FT-IR for molecular structures of TAC films deposited on glass substrate relative to various ratios of blended solutions of aniline and thiophene monomers.

	Wavenumber (cm^−^^1^)	Assignment of FT-IR Absorption Peak
PANI peak	695	meta substitutions, 1, 3 disubstitution in benzene ring
	754	ortho substitutions, 1, 2 disubstitution in benzene ring
	1500	C=C stretching vibration of benzenoid ring
	1610	C=C stretching vibration of quinoid ring
	1676	C=N stretching vibration of quinoid ring
PTh peak	799	C–S stretching vibration
	1400	C=C stretching vibration of thiophene ring
	1676	C=O stretching vibration
	1716	C=O stretching vibration
Common peak	965	C–H out-plane bending of aromatic
	1061	C–H in-plane bending of aromatic
	2888	C–H stretching vibration in CH_2_
	2927	C–H stretching vibration in CH_3_

**Table 3:** Root mean square roughness (Rq) and average roughness (Ra) obtained from AFM images of TAC film surfaces of Figure 4.

Sample	Case I	Case II	Case III	Case IV	Case V
Rq	8.9 nm	58.2 nm	24.1 nm	48.3 nm	59.2 nm
Ra	5.7 nm	42.9 nm	17.5 nm	36.6 nm	44.2 nm

**Table 4:** Elemental compositions of XPS for chemical structure of TAC films deposited on glass substrates by APPJs polymerization technique at two different blending conditions of cases II (aniline 75% and thiophene 25%) and IV (aniline 25% and thiophene 75%).

Conditions	C 1s (Atomic %)	O 1s (Atomic %)	N 1s (Atomic %)	S 2p (Atomic %)
case II	61.5	21.0	4.5	13.0
case IV	62.9	20.6	2.5	14.0

**Table 5:** Peak assignments (BE, eV) and envelope composition (%, total = 100) of C 1s, N 1s, S 2p, and O 1s peaks for chemical structure of TAC films deposited on glass substrates by APPJs polymerization technique at two different blending conditions of cases II (aniline 75% and thiophene 25%) and IV (aniline 25% and thiophene 75%).

Composition of Correlative Functional Group				
Peak Assignment		Composition (%)		
		Binding Energy (eV)	Case II	Case IV
C 1s (%)	C=C	284.1	14.5	10.0
	C–C/C–H	285.0	36.9	37.4
	C–N	285.9	23.8	19.2
	C–O	286.9	16.8	17.7
	C=O	288.1	4.9	13.2
	O–C=O	289.7	3.1	2.5
N 1s (%)	=N–	398.5	22.8	66.5
	–NH–	400.4	51.4	16.3
	=NH^+^	402.1	25.8	17.2
	–NH–/=N–	–	2.3	0.3
S 2p (%)	C–S–C	164.1	68.1	49.5
	C–SO–C	165.7	19.0	38.3
	C–SO_2_–C	168.1	12.9	12.2
O 1s (%)	S=O	531.2	67.2	77.5
	O–C–O	532.6	20.8	14.2
	O=C–O	533.9	12.0	8.3
